# A solitary precaval right renal artery: A case report

**DOI:** 10.1002/ccr3.5866

**Published:** 2022-06-02

**Authors:** Athena Farahzadi, Habibollah Mahmoodzadeh, Nikhil Gopal

**Affiliations:** ^1^ 48439 Fellowship of Surgical Oncology, Cancer Institute Tehran University of Medical Sciences Tehran Iran; ^2^ 48439 Tehran University of Medical Sciences Tehran Iran; ^3^ National Institute of Health National Cancer Institute‐Urologic Oncology Branch Bethesda Maryland USA

**Keywords:** anatomical variation, kidney, precaval renal artery, renal artery

## Abstract

Knowledge of aberrant renal vasculature is critical for preoperative planning of either open or minimally invasive renal surgery. A precaval right renal artery is a rare anatomical variant in which the artery passes anterior to the inferior vena cava(IVC), as opposed to coursing posteriorly. When encountered, this artery is mostly accessory and thus accompanied by renal vessels in orthotopic position. Here, we describe an unusual instance of a solitary, main precaval right renal artery. It can be diagnosed preoperatively by a cross‐sectional imaging study. Awareness of this anomaly is very important to prevent iatrogenic injury during surgical intervention.

## 1 INTRODUCTION

While the incidence of aberrant renal vasculature overall is surprisingly high at 25%, a precaval right renal artery is comparatively infrequent, with rates reported from 0.8%–5%.^1^ The majority of such cases are accessory vessels^2^; however, very rarely, a main right renal artery may course anterior to the inferior vena cava. To our knowledge, only 10 such cases have been described in the literature. Being aware of this anatomical variant in renal procedures is crucial in order to avoid unintentional injury to a major vessel, which can be associated with significant morbidity and/or mortality.

## 2 CASE REPORT

**FIGURE 1:**
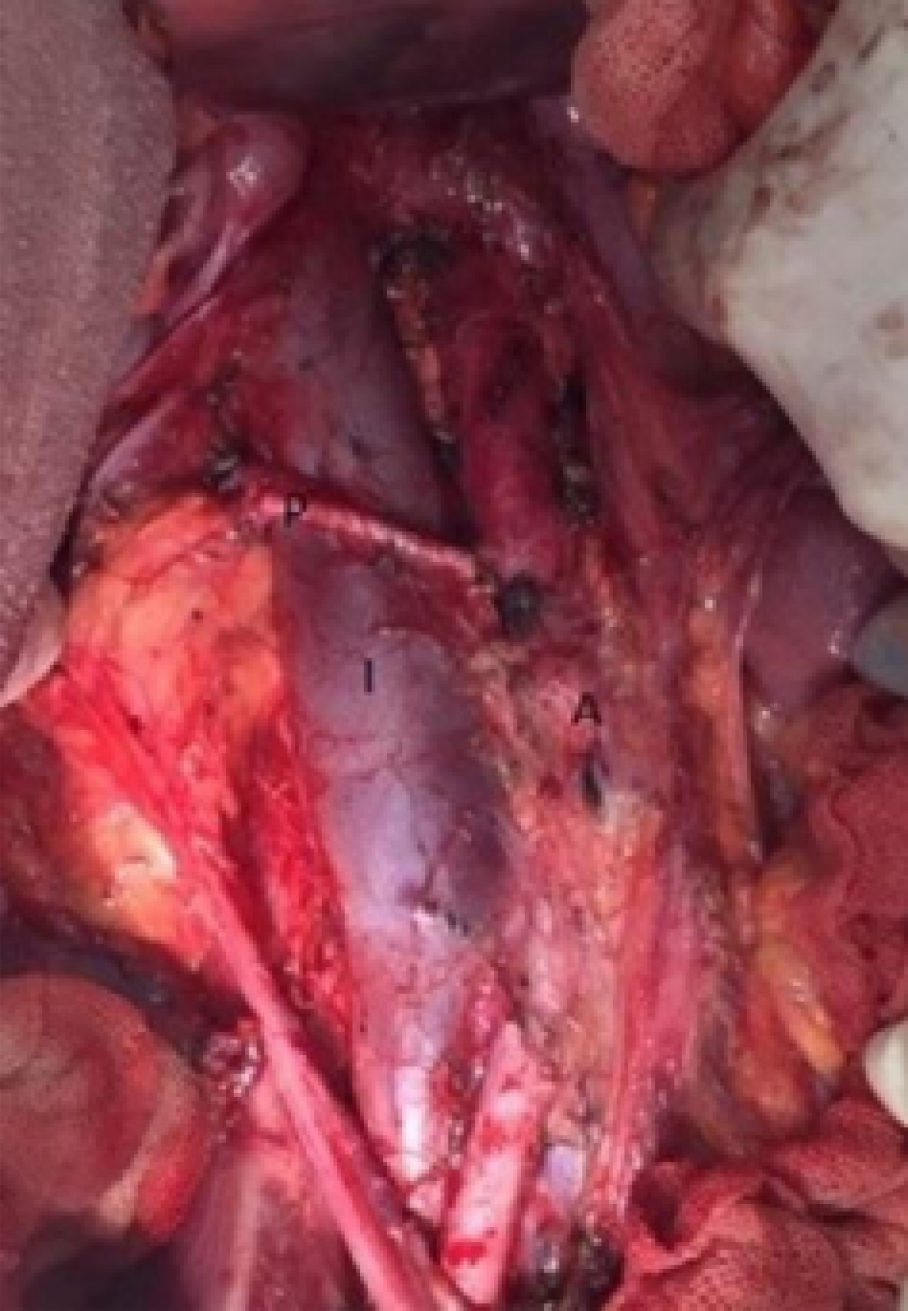
Intraoperative picture showing the right renal artery anterior to the inferior vena cava. I, IVC, A, Aorta, P, Precaval right renal artery

**FIGURE 2:**
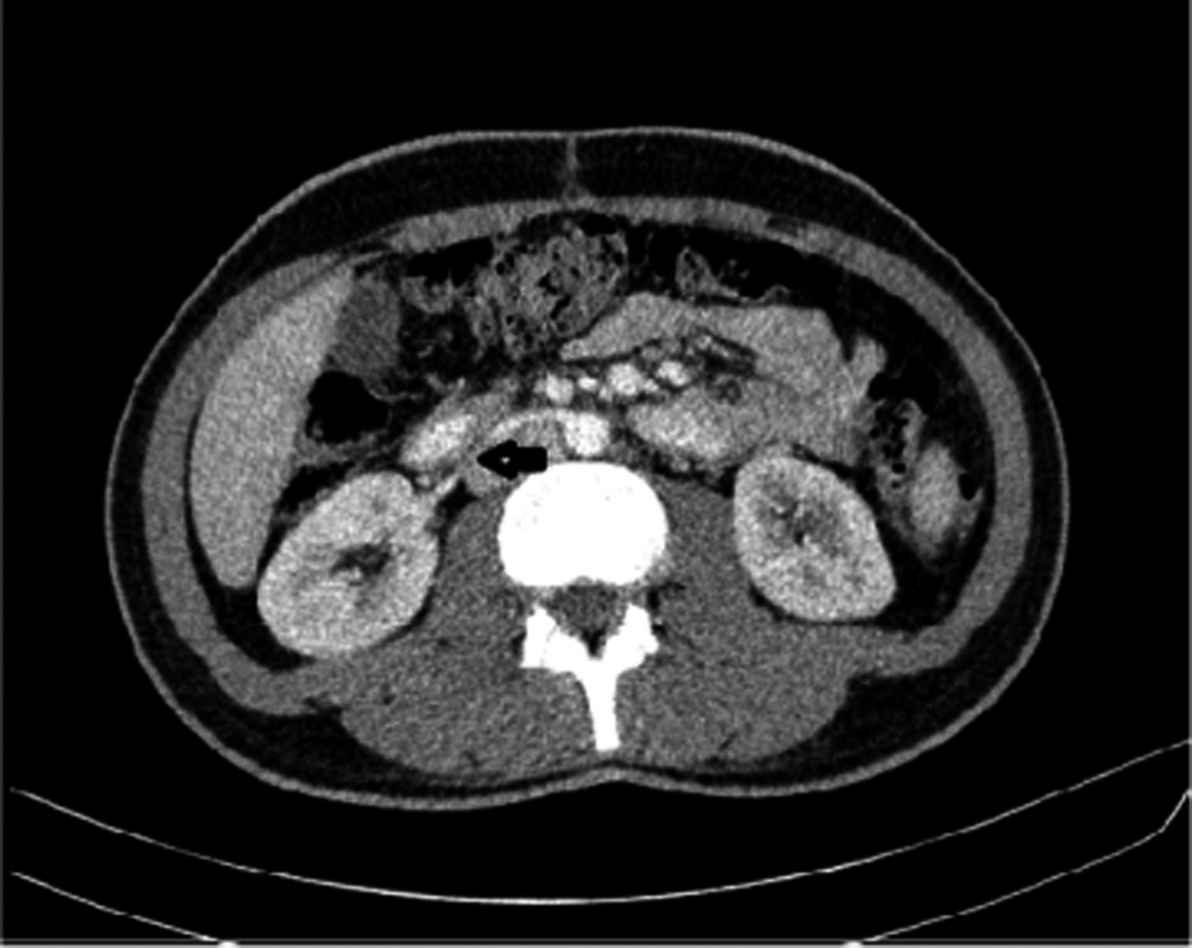
Contrast enhanced Computed tomography scan depicting solitary right renal artery. P, Pre caval right renal artery

A 21‐year‐old man was diagnosed with man was diagnosed with a non‐seminomatous germ cell tumor of the right testis, T2N0Mx, stage 1S. He received two courses of chemotherapy after right orchiectomy. On restaging imaging, he was found to have a perdurable para‐aortic mass measuring about 21.4mm. Subsequent PET scan revealed increased uptake in the para‐aortic area. Additionally, patient was noted to have rising B‐HCG. As this area was the only source of disease identified on imaging, the patient was offered a post‐chemotherapy retroperitoneal lymph node dissection (RPLND). During the operation, he was noted to have a main right renal artery that was anterior to the inferior vena cava (Figure 1). Once the unusual anatomic relationship was noted, care was taken during the paracaval and inter‐aortocaval lymph node dissection to not inadvertently injure the artery. The remainder of the operation proceeded without incident. Afterwards, in reviewing the patient preoperative imaging contrast enhanced CT scan, we defined this aberrant artery (Figure 2). The patient's postoperative course was without any complication. In final histopathology, cancer was noted in 1/65 lymph nodes, with the specific subtype being embryonal carcinoma.

## 3 DISCUSSION

The renal arteries originate from diminution of a series of lateral splanchnic arteries arising from the aorta that form the main blood supply for the mesonephric kidney during embryonic development. This complex pruning of the splanchnic arteries is prone to many variations, which accounts for the relatively common incidence of “aberrant” renal vasculature. The final position of the kidney during migration from the pelvis to lumbar region defines the position and number of renal arteries.^1^, ^2^, ^3^, ^4^ There is good correlation between variants in renal vasculature and ectopic kidney. The incidence rate of renal anomalies in this group is about 20%–30%. The pelvic kidney vasculature is so variable as a result of remaining of its embryological blood supply.^5^

The prevalence of precaval right renal artery has been reported between 0.8% and 5%. The wide variation in prevalence rates may be due to distinct study populations (i.e., racial differences) and/or differences in study design. According to Petit P [6], most of precaval right renal arteries were single and dominant, while Yeh et al proposed that most of precaval right renal arteries were accessory lower pole arteries. Later, Anupma Gupta et al reported in their study that 3% of precaval right renal arteries were dominant and 3% were accessory.^4^, ^6^

A solitary dominant precaval right renal artery(RRA) is a rare occurrence, with about 10 cases described in the literature.^2^, ^7^

The origin of a precaval RRA may be from a persistent caudal vessel originating anteriorly from the aorta after formation of the inferior vena cava, but before the descent of the gonad [8] In addition to a precaval RRA being more easily iatrogenically injured (i.e., during endopyelotomy) or more easily mistaken with other vessels such as the mesenteric or gonadal artery, it can be one of the causes for UPJ obstruction. Therefore, awareness of their existence, ideally preoperatively, is critical to prevent significant morbidity (e.g., nephrectomy)^1^

Accurate preoperative knowledge of renal vascular anatomy is obligatory for surgical planning. Multi‐detector computed tomography (MDCT) angiography is a fast, reliable, non‐invasive, cost effective, imaging tool for universal appraisal of renal vascular anatomy as it displays precious anatomical details with a general accuracy rate ranging from 89% to 100%. MR angiography can rapidly and accurately show renovascular diseases without using contrast medium and ionizing radiation. Therefore, CTA and MRA are regarded to be non‐invasive and powerful diagnostic modalities to evaluate an aberrant renal artery; however, we can define this variant in our case with contrast enhanced CT scan.^8^, ^9^, ^10^

Ignorance of renal vascular variants in any surgical technique may lead to a fatal outcome, particularly in laparoscopic and robotic procedures. Awareness of the number, direction, and connections of the renal arteries is fundamental for accurate planning of surgical procedures involving the kidney, aorta, and retroperitoneal region.^11^

In conclusion, we illustrate an unusual case of a solitary, main precaval right renal artery. The relatively high incidence of renal vascular malformations, including this anomaly, points to the importance of a meticulous preoperative radiological evaluation. Awareness of this anatomical variant is important to prevent inadvertent injury to the renal artery that would lead to potential significant morbidities like nephrectomy, hypovolemic shock from blood loss.

## AUTHOR CONTRIBUTION

All authors were involved in data collection, interpretation, drafting the article, revision of the manuscript, and the final approval of the version to be published.

## CONFLICT OF INTEREST

The authors declare that they have no conflict of interest.

## ETHICAL APPROVAL

Written informed consent was obtained from the patient to publish this report in accordance with the journal's patient consent policy.

## CONSENT

Written informed consent was obtained from the patient to publish this report in accordance with the journal's patient consent policy.
